# Compressive Sensing with Optical Chaos

**DOI:** 10.1038/srep35206

**Published:** 2016-12-02

**Authors:** D. Rontani, D. Choi, C.-Y. Chang, A. Locquet, D. S. Citrin

**Affiliations:** 1 OPTEL Research Group, LMOPS EA 4423 Lab, CentraleSupélec, Université Paris-Saclay, F-57070 Metz, France; 2 LMOPS EA-4423 Lab, CentraleSupélec et Université de Lorraine, F-57070 Metz, France; 3 School of Electrical and Computer Engineering, Georgia Institute of Technology, Atlanta, Georgia 30332-0250, USA; 4 UMI 2958 Georgia Tech-CNRS, Georgia Tech Lorraine, F-57070 Metz, France; 5 School of Physics, Georgia Institute of Technology, Atlanta, Georgia 30332-0250, USA

## Abstract

Compressive sensing (CS) is a technique to sample a sparse signal below the Nyquist-Shannon limit, yet still enabling its reconstruction. As such, CS permits an extremely parsimonious way to store and transmit large and important classes of signals and images that would be far more data intensive should they be sampled following the prescription of the Nyquist-Shannon theorem. CS has found applications as diverse as seismology and biomedical imaging. In this work, we use actual optical signals generated from temporal intensity chaos from external-cavity semiconductor lasers (ECSL) to construct the sensing matrix that is employed to compress a sparse signal. The chaotic time series produced having their relevant dynamics on the 100 ps timescale, our results open the way to ultrahigh-speed compression of sparse signals.

Compressive sensing (CS) defines a novel mathematical framework combining sampling and compression. This enables the sampling of band-limited analog signals below the lower-bound rate set by the Nyquist-Shannon theorem. Under such sub-sampling conditions, recent theoretical work^1^,^2^ has shown that *exact* reconstruction of sub-sampled data is possible under the assumption of sparsity, which refers to concise information content in a signal with respect to its occupied bandwidth. Applications of this new framework have already permeated various areas of physics and engineering such as reconstruction of three-dimensional images with limited number of sensors^3^, reconstruction of quantum states via compressive tomography^4^, and prediction of dynamical network structures from limited data^5^.

In CS, the measurement vector **y** of the sparse signal **x** is realized via the *sensing matrix* Φ such that **y** = Φ**x**. This linear operation generates an incomplete set of information representative of the sparse signal that is then used to reconstruct the information via a minimization problem with respect to the 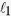-norm (*i.e.* sum of absolute values of each vector component). This method works optimally if the sensing matrix satisfies the restricted isometry property (RIP), which means mathematically that all subsets (with a size dependent on the level of sparsity considered) of columns in the sensing matrix are nearly orthogonal^6^,^7^.

Hence, generating sensing matrices with RIP is critical for achieving an optimal reconstruction of sparse signals with CS. It has been shown that randomness can lead to effective sensing mechanisms^7^. For example, using Gaussian or Bernouilli random variables as elements of the sensing matrix will guarantee mathematically proven RIP^1^. Other studies have also demonstrated that deterministic sensing matrices could be generated using advanced coding techniques^8^,^9^.

Recently, Yu *et al*. have proposed the use of chaotic sequences generated by a logistic map to populate sensing matrices and have demonstrated mathematically that RIP was achieved with high probability for a level of performance comparable to those generated with Gaussian random variables^10^. The interest in chaotic systems for CS relies on their broadband (tens of GHz) noise-like features and their potential hardware implementation with physical devices.

External-cavity semiconductor lasers (ECSL) are known for generating large-bandwith chaos used in many applications^11^,^12^, such as secure communications^13^,^14^ and ultrafast generation of true random numbers^15^,^16^. Nevertheless, the presence of the time delay in the dynamics induces temporally nonlocal correlations^17^ that could potentially hinder the level of performance of CS. Furthermore, the complexity of the optical chaos generated by an ECSL strongly depends on the choice of operating parameters (pumping current, feedback strength, and time delay)^18^ and also presents interesting scaling properties^19^,^20^ that could be useful for predicting parameter regimes, for which a chaotic sensing matrice has the RIP property empirically.

In this study, we demonstrate experimentally for the first time the possibility to harness optical chaos generated by an ECSL for the generation of a sensing matrix with an optimal level of performance for high-speed CS. We unveil the existence of a wide parameter range that allows for the reconstruction of one-dimensional sparse signals (known as the *basis pursuit* problem) and image reconstruction. We also find that despite being statistically non-Gaussian and strongly correlated for fast sampling rate, optical chaos guarantees similar performance in terms of reconstruction error and level of maximum sparsity.

Our experimental setup is described in Fig. 1(a). It consists of a single-longitudinal-mode DFB laser diode emitting at 1550 nm based on a InGaAsP quantum well for the gain medium. The threshold current for free-running operation is *J*_*th*_ = 9.27 mA and the maximum cw power is 15 mW. The external cavity introduces a time delay ~4.3 ns and is comprised by a mirror (M) mounted on a translation state (TS), a polarizer (P) and a quarter wave plate (QWP), whose angle relative to the polarization direction of light is controlled by a piezoelectric actuator. The QWP angle is used to control the optical feedback strength *η*, which reaches up to ~20% of optical power, not taking into account coupling losses into the laser. A beam splitter (BS) is introduced to build a measurement arm that comprises an optical isolator (IO). A 12-GHz amplified photodiode (PD) NewFocus 1544-B, and a broadband oscilloscope Agilent DSO 80804B with 12 GHz bandwidth and 40 GSa/s sampling rate are employed.

As described in Fig. 1(b), the oscilloscope samples the intensity time series *I* (*t*) = |*E(t*)|^2^ with period Δ*t* = 25 ps and quantizes it with 8-bits precision to produce voltage *V (t*) ∝ *I (t*). The digitized chaotic time series is then sub-sampled by a factor *d* greater than the maximum decorrelation time of the chaotic intensity time series to ensure that two consecutive samples are maximally decorrelated. This guarantees approximate independent and identical distribution (iid) of the samples used to build the sensing matrix, but we will later show that this constraint can be relaxed in the construction of chaotic sensing matrices. We retain *M* × *N* samples 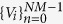 with *V*_*i*_ = *V* (*t*_*i*_ = *t*_0_ + *id*Δ*t*). Then, we form the chaotic intensity sensing matrix 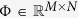 as


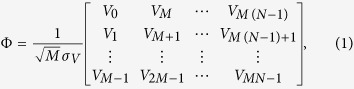


with *σ*_*V*_ the standard deviation of the chaotic optical intensity measured. The construction of this matrix follows similar principles to those of ref. 10.

We first analyze the statistical properties of three typical experimental chaotic time series of different pumping currents and feedback rates corresponding to dynamical regimes ranging from weakly to fully developed coherence collapse^21^. We observe in Fig. 2(a) that the probability density functions (pdf) for each set of parameters have significantly different shapes, which may potentially impact the performance of CS. For example, at weak feedback rate *η* and pumping current *J* relatively close to threshold *J*_*th*_, the distribution is sharpely peaked whereas at high pumping current the distribution is flattened with an approximate four-fold variance increase. For the various distributions, we have performed a one-sample Kolmogorov-Smirnov test guaranteeing with a 5% significance level that optical chaos is not sampled from a Gaussian distribution.

We also analyze in Fig. 2(b) the spectral properties of the chaotic time series by computing the normalized autocorrelation function (ACF) defined mathematically by 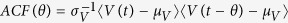 with 〈·〉 the time average, *θ* a time lag, and *μ*_*V*_ and *σ*_*V*_ the mean and standard deviation of the voltage time series *V (t*), respectively. We zoom in the vicinity of the zero time-lag and observe oscillations reminiscent of the relaxation-oscillation dynamics of the laser diode^16^. For time lags greater than 25Δ*t*, the correlation has pratically vanished retaining very little of the relaxation oscillation influence for the sets of parameters investigated. For 50Δ*t*, the correlation has completely vanished thus making *d* = 50 a suitable default sub-sampling rate to create a sensing matrix Φ with RIP.

Next, to demonstrate CS with optical chaos, we consider the one-dimensional basis pursuit (BP) problem. We generate a time-sparse signal 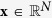 comprising only *K* < *N* randomly distributed non-zero spikes and with amplitude also randomly chosen to be equal to ±1 with probability 0.5. We construct the measurement vector 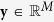, **y** = Φ**x** to be used for the signal reconstruction. CS gives an accurate reconstruction of the sub-sampled data by solving the following linear-programming optimization problem:


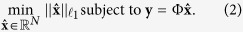


In our numerical analysis, we solve Eq. (2) with the 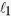-Magic Software Toolbox^22^. In the following, we use a similar benchmark to those employed in ref. 10 and observe the influence of the ECSL operational parameters.

Success in solving the BP problem with CS is given in a probabilistic sense with respect to the 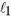-norm. Hence, we first characterize the recovery rate of time-sparse signals for fixed size *N*. This is defined as the probability that the relative error of reconstruction with respect to the 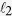-norm is below a given bound and is expressed mathematically as 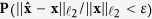 with *ε* = 0.01. We generate one hundred instances of time-sparse signals of size *N* = 100 for each increasing value of sparsity level *K* ∈ [0, 40] and ten instances of the sensing matrix Φ leading to a measurement vector of size *M* = 50. We use this approach to obtain meaningful probability estimations.

Figure 3(a) shows the recovery rates using a chaotic sensing matrix obtained with optical chaos with pumping current *J*_2_ = 1.83*J*_*th*_, feedback rate *η*_2_ = 12% of the light fed back, and sub-sampling factor *d* = 50. The curve has an S-shape with two plateaus corresponding to quasi-perfect and failed reconstruction with probability one, respectively. This is compared to the recovery rate (in red) obtained using Gaussian random variables, which is known as providing asymptotically optimal reconstruction performance. We notice that the two curves are practically superimposed, hence showing that optical chaos can provide an optimal level of performance for the BP problem in CS and result in the sensing matrices having the RIP. As an illustration, we show in Fig. 3(b–c) examples of perfect and failed reconstruction of one-dimensional sparse signals associated to the various level of sparsity at constant signal and observation sizes.

To gain additional insight on the performance of chaos-based sensing, we analyze the recovery rate as a function of both the number of measurements and sparsity level. In Fig. 4(a), we compute the recovery rate, with an identical threshold to that of Fig. 3(a), in the plane (*γ* = (*N* − *M*)/*N, ε* = *K/N*) and with sparse signal of size *N* = 100. For each couple of measurement-sparsity values, we perform 1000 numerical experiments to estimate the probability of sucessful recovery. Similar to ref. 23, we observe a sharp and rapid transition from a recovery rate approximately equal to 1 to zero, which is also known as the *Donoho-Tanner barrier*. To compare the relative performance of optical chaos-based sensing and Gaussian sensing, we display in Fig. 4(b) the difference in recovery rates. We observe that the two sensing mechanisms lead to similar levels of performance for the BP problem. Specifically, the only differences, in the entire plane (*γ, ε*), occur in the phase transition, where small statistical fluctuations of magnitude ±0.04 are observed.

For practical concerns, investigating the impact of operational parameters such as pumping current and optical feedback strength is key to ensure the use of optical chaos robustly for CS applications. Figure 5(a) shows the recovery rate of the BP problem for seven different feedback strengths corresponding to a different dynamical complexity for the optical chaos. (This can be loosely quantified by the amplitude of the autocorrelation peak *ACF* (*θ* = *τ*) at the delay of the external cavity *τ* ~ 4.3 ns.) The range of possible recovery rates (shown by error bars) is maximally bound by ±0.020 and hence shows that a wide interval of feedback strengths provides performance similar to those of Gaussian-based sensing. The influence of pumping current (not shown) leads to similar results with errors bars bounded by ±0.038. We have also quantified the maximum level of fluctuation in recovery rate for a fixed set of operational parameters for the chaotic laser (identical to that of Fig. 3) and for one hundred realizations of the chaotic sensing matrices. We found that the fluctuations are bounded by ±0.027, thus demonstrating the robustness of the experimental generation of chaotic sensing matrices that provide a quasi-optimal level of performance.

Another parameter to investigate is the sub-sampling factor *d*, which sets an upper bound on the maximum achievable rate for the exploitation of optical chaos in CS. For certain applications, such as random number generation, it is critical to remove temporal correlations to pass statistical tests^15^. In CS theory, the sensing matrix should be constructed with iid random variables, hence suggesting that the correlation between optical chaos samples should be negligible as well. However, constructing the sensing matrix Φ columnwise allows one to relax this condition without impeding the level of performance^24^.

Figure 5(b) shows that sub-sampling factors smaller than 50 still allow for optimal performance, despite a significant increase of correlation level. According to our analysis, even using unitary sub-sampling *d* = 1 corresponding to a correlation level greater than 0.6 does not result in a noticeable loss of performance on the BP problem. The range of recovery rate (given by error bars) is bounded by ±0.024 and the recovery curve is still well surimposed with the optimal curve. Hence, the difference between recovery rate for correlated and uncorrelated chaotic samples both lead to a sensing matrix Φ with empirical RIP. This observation corroborates simulations realized with correlated Gaussian iid variables to build sensing matrices^24^.

An additional interest of our approach is that optical chaos can be readily exploited for CS without any additional post-processing, while maintaining an optimal level of performance. This simplifies its potential use in information processing architectures and is in clear contrast with the more common use of chaotic lasers as energy-efficient and ultra-fast random-number generators^11^,^25^. Indeed, this type of application usually requires significant post-processing and sometimes even discarding some of the most significant bits resulting from the ADC process^26^ in order to pass standard randomness tests^27^,^28^, although recent progress has allowed to keep more bits from the sampled waveform^29^.

Finally, we use the chaotic sensing matrix in a CS problem consisting of reconstructing a 2D image based on a limited measurement rate. As defined by the CS framework, we need to introduce a sparsifying matrix 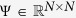 realizing a change of basis [*e.g.* the wavelet transform]. To reconstruct an image, we modifed the linear program of Eq. (2) to


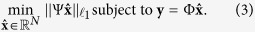


This linear program is searching for a sparse solution in the sparse basis leading to the obtained measurements. We consider the Shepp-Logan phantom image^30^ with *N* = 4096 pixels shown in Fig. 6(a) as the original data to be reconstructed using CS. We use a two-level bidimensional discrete wavelet decomposition with Daubechies wavelets for our sparsifying transformation [see Fig. 6(b)]. We consider a measurement rate of 0.4 (*M* = 1638) and a chaotic sensing matrix with sub-sampling factor *d* = 1 and (*J*_2_, *η*_2_) as operating parameters. Finally, we solve Eq. (3) using the 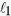-Magic Software Toolbox. The recovered image is shown in Fig. 6(c) and displays the main features of the original data despite imperfections [see Fig. 6(d)] due to the sparsity level (*K* = 1988) relative to the number of measurements *M*. The quality of the image is quantified using the peak-signal-to-noise ratio 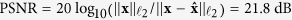, which denotes an accurate reconstruction. It is possible to achieve higher SNR using different linear programs, such as the TV Algorithm^1^, tailored for certain image constructions where the image gradient is sparse (which is the case of the Shepp-Logan phantom image).

As a perspective, we envision that such a chaotic laser could replace the pseudo-random number generator based on an linear-feedback shift register (LFSR) which is traditionally used in the so-called random premodulation integrator (RMPI) scheme^31^. In the original RMPI approach, a LFSR generates binary random numbers that are mixed with the analog signal of interest. The mixed signal is then integrated and sampled at a rate slower than the Nyquist rate. To make use of optical chaos in RMPI, one would need to preprocess the optical chaotic carrier to make it a piecewise-constant signal prior to the analog mixing with the signal of interest. This operation could be realized electronically. The resulting chaotic piecewise-constant signal could be then back converted into an analog signal to be mixed with the signal of interest. Being piecewise-constant allows for a countable number of values to populate the sensing matrix and makes the compressive-sensing reconstruction computationally tractable.

In this study, we have demonstrated experimentally that chaotic time series generated by a laser diode with optical feedback can be used for compressive sensing applications. Using the benchmark of the basis pursuit problem and reconstruction of the Shepp-Logan phantom image, we demonstrated that sensing matrices built with samples from the chaotic time series lead to very good and robust reconstruction of one-dimensional sparse signals even in the presence of a high level of correlation between consecutive samples. This opens a new field of application for optical chaos in ultra-fast information processing.

## Additional Information

**How to cite this article**: Rontani, D. *et al*. Compressive Sensing with Optical Chaos. *Sci. Rep.* **6**, 35206; doi: 10.1038/srep35206 (2016).

**Publisher's note:** Springer Nature remains neutral with regard to jurisdictional claims in published maps and institutional affiliations.

## Figures and Tables

**Figure 1:**
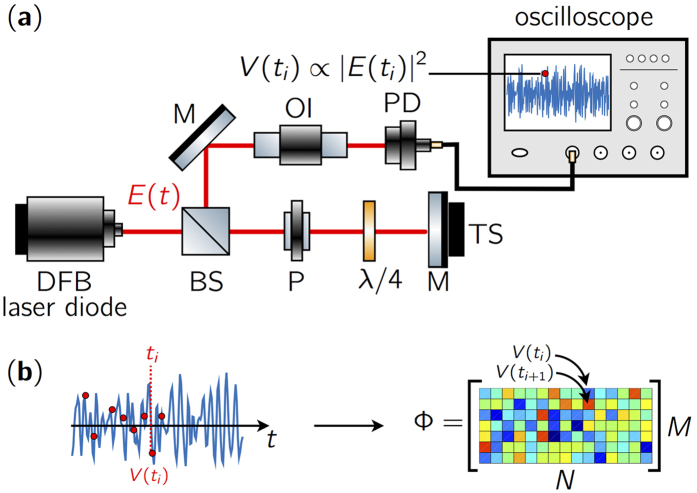
(**a**) Illustration of the experimental setup of an external-cavity semiconductor laser (ECSL) generating a chaotic electric field *E* (*t*). The intensity *I* (*t*) = |*E* (*t*)|^2^ is detected by the photodiode and converted into voltage *V (t*). (**b**) Digitization with 8-bit precision and subsampling (*t*_*i*_ = *id*Δ*t* with 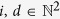) of the voltage time series *V (t*) for the construction of the sensing matrix Φ. Consecutive samples are placed within the same column.

**Figure 2:**
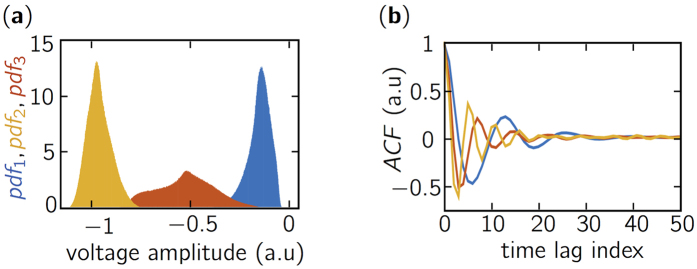
(**a**) Estimation of probability density functions (*pdf*_1,2,3_) of the optical chaos detected by the photodiode for *J*_1_ = 1.19*J*_*th*_, *η*_1_ = 5%, *J*_2_ = 1.83*J*_*th*_, *η*_2_ = 12% and *J*_3_ = 2.59*J*_*th*_, *η*_3_ = 20%, respectively. They are represented by blue, yellow, and red solid lines. (**b**) Normalized autocorrelation function close to the zero time lag for identical experimental conditions and choice of representation.

**Figure 3:**
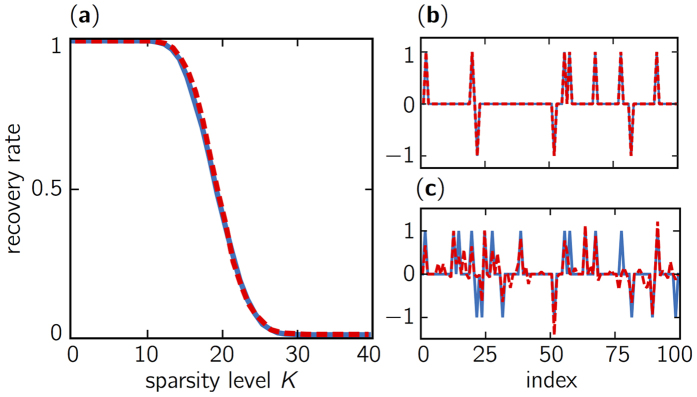
(**a**) Recovery rate curves for the one-dimensional BP problem. The solution with a chaotic sensing matrix is given by the blue solid line and the optimal solution given by a Gaussian random sensing matrix is in red dashed line. Curves are obtained over a 1000 trials to compute the probability estimate. (**b**) Example of perfect reconstruction with unitary probability for low sparsity signal (*K* = 10). (**c**) Example of imperfect reconstruction occuring with probability 1/2 for signal with a higher sparsity level (*K* = 20). Original and reconstructed signals are represented by a blue solid and red dashed lines, respectively.

**Figure 4:**
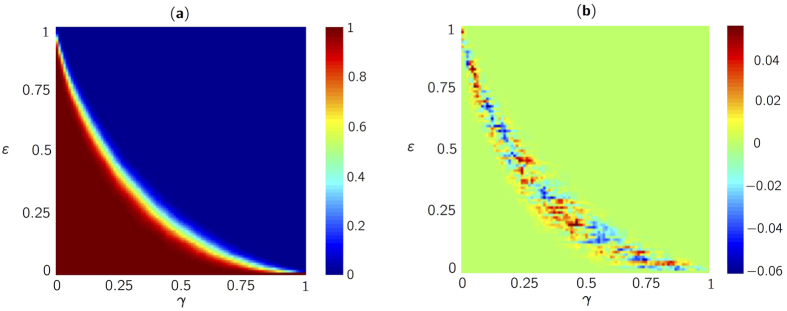
(**a**) Recovery map illustrating the Donoho-Tanner barrier for the chaos-based sensing matrix construced column-wise for the BP problem obtained in the normalized measurement and sparsity plane (*γ* = (*N* − *M*)/*N, ε* = *K/N*) for feedback strength *η* ≈ 12%, pumping current *J* = 1.83*J*_*th*_ and sub-sampling rate *d* = 50. (**b**) Recovery map for the difference between Gaussian and chaos-based sensing.

**Figure 5:**
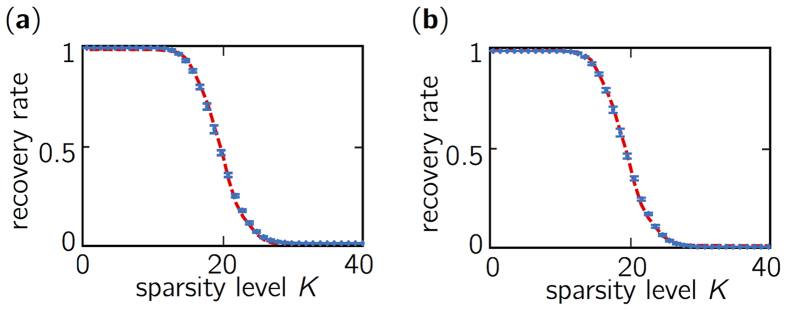
Recovery rate curves for the BP problem obtained (**a**) for multiple feedback strengths (controlled by the polarizer angle) *η* ≈ 0.05, 0.6, 2.5, 5, 8.5, 12, 20 in percent of the light fed back at the facet of the laser diode, pumping current *J*_2_ and sub-sampling rate *d* = 50; (**b**) for various sub-sampling factors *d* = 1, 2, 5, 10, 25, 50 and for other parameters (*J*_2_, *η*_2_). The error bars (in blue) correspond to the range of recovery rates with chaotic sensing matrices. The reference curve obtained with Gaussian random variable is in dashed red.

**Figure 6:**
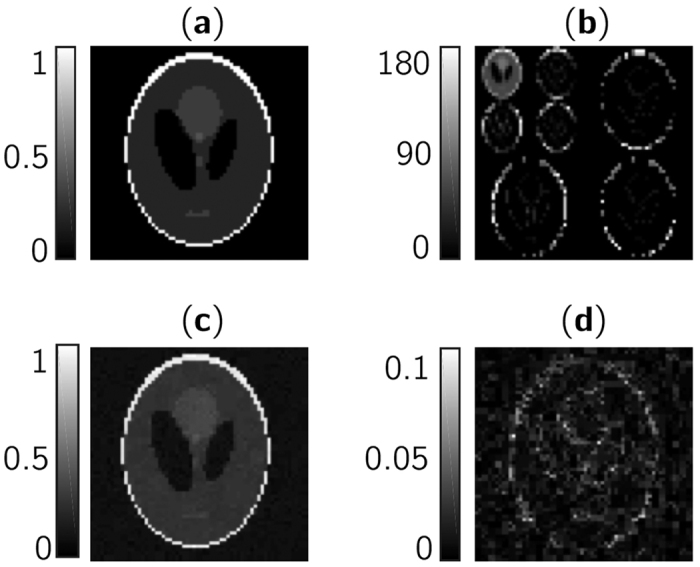
Reconstruction by compressive sensing of an image with a chaotic sensig matrix. (**a**) Original Shepp-Logan phantom image with 64 × 64 pixels. (**b**) Representation of the image in the wavelet domain with two scaling levels, where the reconstruction is performed. (**c**) Recovered image by compressive sensing with measurement rate 0.4 and a chaotic sensing matrix. (**d**) Absolute value of the residual error from imperfect reconstruction.
